# What Should Be the Training for Cottage Hospital Matrons?

**Published:** 1886-10-09

**Authors:** K. M. Heanley


### WHAT SHOULD BE THE TRAINING FOR COTTAGE HOSPITAL MATRONS?

It seems to me this subject is generally misunderstood, because it is believed that little is required. This is to be regretted, because the position really makes large demands upon the lady holding such an office, because in hospitals of eighteen or twenty beds there is generally no resident house-surgeon, and it is not always easy to obtain an honorary officer at a short notice. Hence the matrons of such hospitals ought to be equal to any emergencies. They ought also to have a knowledge of dispensing and testing; indeed, not less, but more practical knowledge should be expected of them than of the matron of a large hospital. An opinion on this subject coming from you would have weight, and produce an influence on the training for such posts which would react favourably on the institutions themselves.

Have you succeeded in establishing any nurses' savings, or sick fund, and if so, will you let me have the particulars?

Believe me, yours obliged,

K. M. Heanley.

*** We shall be glad to have further letters on the training of cottage hospital matrons. The pension-fund scheme is being carefully elaborated, and more will, we understand, be heard of it in a few months' time.—Ed. T. H.

